# Human seroprevalence indicating hantavirus infections in tropical rainforests of Côte d’Ivoire and Democratic Republic of Congo

**DOI:** 10.3389/fmicb.2015.00518

**Published:** 2015-05-21

**Authors:** Peter T. Witkowski, Siv A. J. Leendertz, Brita Auste, Chantal Akoua-Koffi, Grit Schubert, Boris Klempa, Jean-Jacques Muyembe-Tamfum, Stomy Karhemere, Fabian H. Leendertz, Detlev H. Krüger

**Affiliations:** ^1^Institute of Virology, Helmut-Ruska-Haus, Charité Medical SchoolBerlin, Germany; ^2^P3 – Epidemiology of Highly Pathogenic Viruses, Robert Koch InstituteBerlin, Germany; ^3^Université Alassane Ouattara de BouakéBouaké, Côte d’Ivoire; ^4^Institute of Virology, Slovak Academy of SciencesBratislava, Slovakia; ^5^National Institute of Biomedical ResearchKinshasa, Democratic Republic of Congo

**Keywords:** hantavirus, Africa, seroprevalence, Côte d’Ivoire, Democratic Republic of Congo, tropical rain forest

## Abstract

Hantaviruses are members of the *Bunyaviridae* family carried by small mammals and causing human hemorrhagic fevers worldwide. In Western Africa, where a variety of hemorrhagic fever viruses occurs, indigenous hantaviruses have been molecularly found in animal reservoirs such as rodents, shrews, and bats since 2006. To investigate the human contact to hantaviruses carried by these hosts and to assess the public health relevance of hantaviruses for humans living in the tropical rainforest regions of Western and Central Africa, we performed a cross-sectional seroprevalence study in the region of Taï National Park in Côte d’Ivoire and the Bandundu region near the Salonga National Park in the Democratic Republic (DR) of Congo. Serum samples were initially screened with enzyme-linked immunosorbent assays using nucleoproteins of several hantaviruses as diagnostic antigens. Positive results were confirmed by Western blotting and immunofluorescence testing. Seroprevalence rates of 3.9% (27/687) and 2.4% (7/295), respectively, were found in the investigated regions in Côte d’Ivoire and the DR Congo. In Côte d’Ivoire, this value was significantly higher than the seroprevalence rates previously reported from the neighboring country Guinea as well as from South Africa. Our study indicates an exposure of humans to hantaviruses in West and Central African tropical rainforest areas. In order to pinpoint the possible existence and frequency of clinical disease caused by hantaviruses in this region of the world, systematic investigations of patients with fever and renal or respiratory symptoms are required.

## Introduction

Hantavirus infections are responsible for human disease worldwide with up to 200,000 cases estimated per year. As the only bunyaviruses being carried by small mammals, these zoonotic agents can cause Hemorrhagic Fever with Renal Syndrome (HFRS) or Hantavirus Cardiopulmonary Syndrome (HCPS) with case fatality rates of up to 50% (Krüger et al., 2011, 2015).

Sangassou virus as the first autochthonous African hantavirus was found in Guinea, West Africa, in 2006. First serological studies of patients with fever from the area indicated human hantavirus infections implying underestimation of hantavirus public health impact on the African continent (Klempa et al., 2006, 2010, 2012). During the recent years a high number of novel hantaviruses from non-conventional hosts, as shrews, moles, and bats, have been molecularly detected, many of them in Africa (for a review, see Witkowski et al., 2014). The mere existence of these vastly uncharacterized viruses induces speculations about their emergence in further hosts and geographic areas. Particularly in Africa, where many diseases remain neglected or underreported due to bad health care conditions, and other hemorrhagic fever viruses like Lassa or Ebola occur, nearly nothing is known about the epidemiology and case definition of hantavirus disease in humans. The typical clinical symptoms known from hantavirus-infected patients in the Old and New World include high fever, hemorrhages, thrombocytopenia, abdominal pain, flu-like symptoms, and finally organ failure – symptoms known to be also caused by many other hemorrhagic fever viruses including arena-, filo-, and flaviviruses (Kortepeter et al., 2011; Heinz and Stiasny, 2012; McLay et al., 2014). Recent outbreaks of zoonotic diseases, like MERS coronavirus on the Arabian Peninsula (de Groot et al., 2013) or Ebola virus in West Africa (Baize et al., 2014), demonstrate a high necessity for intensive studies of highly pathogenic zoonotic viruses in order to be prepared for their emergence and major outbreaks.

Here we investigated the presence of hantavirus antibodies in humans living in the Taï National Park in Côte d’Ivoire as part of the West African Guinean Forest Block and of the Salonga National Park in the Democratic Republic (DR) of Congo as part of the Central African Congo Basin – two regions where the richest and oldest tropical evergreen rainforest biomes on Earth exist (Jones, 1994), potentially facilitating close contact and high exposure of the human population to putative hantavirus hosts. So-called bush meat, which includes small mammals such as bats and rodents, is consumed regularly by inhabitants of the villages in scope. It seems obvious that living within or close to a biodiversity hotspot, like a tropical forest, leads to high risk of infection by a variety of infectious agents (Wolfe et al., 2007; Keesing et al., 2010). While indigenous hantaviruses have already been found in animal samples from Côte d’Ivoire [Azagny virus (Kang et al., 2011), Mouyassué virus (Sumibcay et al., 2012)], DR Congo thus far remains a country without proven hantavirus occurrence. Nevertheless, bat species shown to carry hantaviruses elsewhere (Sumibcay et al., 2012; Weiss et al., 2012) naturally occur in the region.

## Materials and Methods

### Study Sites and Populations

**FIGURE 1:**
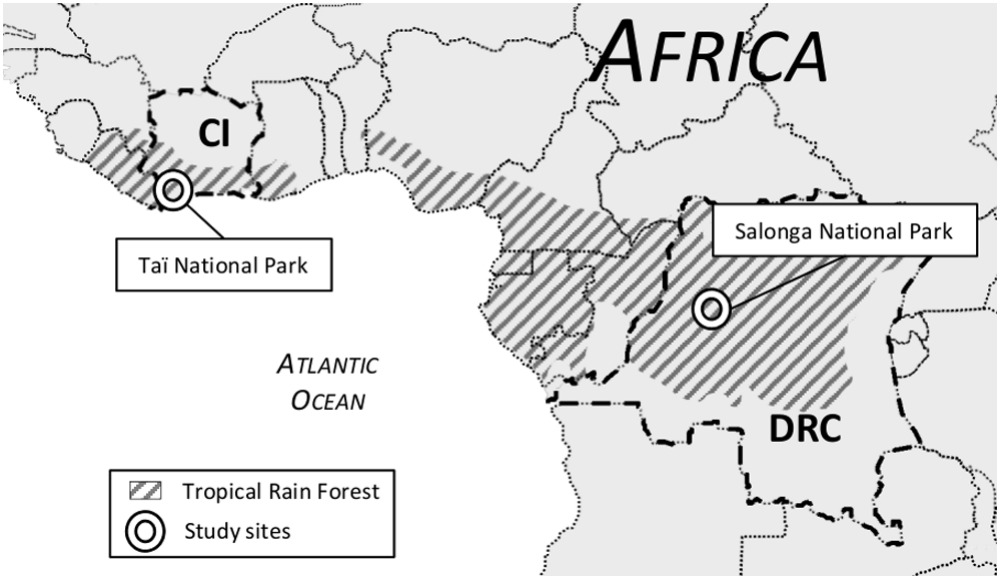
**Map of Western/Central Africa showing location of study sites illustrated by circles.** The shaded areas mark tropical rain forest biome. CI, Côte d’Ivoire; DRC, Democratic Republic of Congo.

To screen for seropositive individuals, serum samples were collected from 16 villages in Taï National Park of Côte d’Ivoire in 2007 and five villages in Salonga National Park of DR Congo in 2011 (**Figure 1**). The investigation was approved by the National Ethics Commissions of both countries and all participants gave their approval prior to the sampling. The sera were frozen directly in the field in liquid nitrogen. Altogether 982 samples were analyzed – for sample composition according to location, see **Table 1**. Participants were also asked for professional status to estimate possible virus exposure and for recent health problems.

**Table 1:** Sample composition and proof of antibody-positive sera according to sampling location.

Village	Country	Seropositives/no. tested
Daobly	CI	0/34
Djereoula	CI	0/31
Djidoubaye	CI	1/38 (2.6%)
Gahably	CI	0/54
Goulegui Beoue	CI	1/54 (1.9%)
Keibly	CI	7/88 (8.0%)
Ponan	CI	0/18
Portgentil	CI	1/47 (2.1%)
Sakre	CI	6/73 (8.2%)
Sioblooula	CI	3/34 (8.8%)
Taï	CI	0/26
Tieleoula	CI	2/36 (5.6%)
Tienkoula	CI	2/39 (5.5%)
Zagne	CI	0/12
Zaipobly	CI	3/35 (8.6%)
Ziriglo	CI	1/68 (1.5%)
***Total CI***		***27/687 (3.9%)***
Bekombo	DRC	0/3
Ipope	DRC	1/57 (1.8%)
Iyoko	DRC	0/1
Lompole	DRC	4/134 (3.0%)
Nganda	DRC	2/100 (2.0%)
***Total DRC***		***7/295 (2.4%)***

CI, Côte d’Ivoire; DRC, Democratic Republic of Congo.

### Serological Test Algorithm

The collected sera were analyzed following a serodiagnostic algorithm as described previously (Klempa et al., 2010; Witkowski et al., 2014). Shortly, a screening enzyme-linked immunosorbent assay (ELISA) was performed using recombinant nucleocapsid antigens from prototypical hantaviruses including Dobrava-Belgrade virus, Puumala virus (Meisel et al., 2006), and the African Sangassou virus with a serum dilution of 1:200. All positive samples were subsequently tested in an in-house Western Blot (WB) with Sangassou virus antigen and by recomLine HantaPlus strip blot (Mikrogen, Germany) according to manufacturer’s specifications with a dilution of 1:100. Finally, an in-house immunofluorescence assay (IFA) with VERO-E6 cells infected with Hantaan, Dobrava-Belgrade, Puumala, or Sangassou viruses was performed. The lowest dilution for IFA was 1:20, followed by titration of samples with sufficient volumes available. Sera positive in all three test formats were considered as hantavirus seropositive. Focus reduction neutralization assay (FRNT) against Dobrava-Belgrade, Puumala, and Sangassou viruses was performed for selected samples, with enough sample material.

### Molecular Testing

Samples with high absorbance values in ELISA were also tested for hantavirus IgM antibodies and, in case of a positive result, RNA was extracted and screened by pan-hantavirus RT-PCR (Klempa et al., 2006) to target possible current infections which exhibit RNAemia.

### Statistical Analyses

The database with serodiagnostic results was established in Excel® for Windows, before being transferred to Stata (Stata/SE12.0 for Windows, Stata Corp, College Station, TX, USA). Seroprevalences were calculated including 95% confidence intervals (CI). The effect of country, sex, age, village, and occupation on the serological status was initially investigated with logistic regression models with serological status as binary dependent variable and the other factors as independent variables.

## Results

### Samples and Demographic Data

A total of 982 blood samples were analyzed in the study, 687 and 295 samples were collected in Côte d’Ivoire and DR Congo, respectively. The average number of samples per village was 49 (range 1–134). The mean age of the participants was 35 years (range 3–95, *n* = 932). 53% of the participants were females (reported gender, *n* = 953). Occupation data were available for 92% (*n* = 900) of the samples. Most people were occupied with agriculture (40%, *n* = 356) or house work (28%, *n* = 255), 15% (*n* = 134) were pupils and the rest reported the following occupations: teacher/student, sales/business person, cook/restaurant worker, tailor, driver/mechanic, fisherman, builder, hunter/meat seller, and health worker. For sample composition according to profession, age, and gender, see **Table 2**.

**Table 2:** Seropositive participants from both study sites according to profession, age, and gender in both countries.

Analysis by	Seropositives/no. tested
**Profession**
Agriculture	14/356 (3.9%)
House work	9/255 (3.5%)
Pupils	3/134 (2.2%)
Teacher/student	0/27
Sales/business	1/24 (4.2%)
Cook/restaurant	0/19
Tailor	1/17 (5.9%)
Driver/mechanic	0/16
Fisher	1/16 (6.25%)
Various	0/15
Builder	0/9
Hunter/butcher	0/7
Healthcare	0/5
***Total***	**29/900**
**Age**
1–10	1/38 (2.6%)
11–20	7/207 (3.4%)
21–40	14/327 (4.3%)
41–60	5/226 (2.2%)
>60	5/89 (5.6%)
***Total***	***32/932***
**Gender**
Female	19/503 (3.8%)
Male	15/450 (3.3%)
***Total***	***34/953***

Profession and age were known for 900 and 932 participants, respectively.

### Serology and Molecular Testing

The confirmed seroprevalence was 3.9% (27/687; 95% CI 2.6–5.7%) and 2.4% (7/297; 95% CI 1.0–4.8%) for the groups from Côte d’Ivoire and DR Congo, respectively. There was no statistical significant difference between the prevalences in the two countries (*p* > 0.05). The primary screening ELISA detected 229/982 seropositive samples against Sangassou virus antigen and only 28/982 against Puumala virus antigen. Out of all samples found to be positive in the screening ELISAs, 34 sera were confirmed to be seropositive by all levels of the test algorithm. Thirty out of the 34 seropositive samples were found to be reactive (12 of them even exclusively) against Sangassou virus antigen in the screening ELISA. For detailed results, see Supplementary Table S1. For 13/34 of the positive sera, where FRNT was performed, none of the used viruses was neutralized effectively (data not shown).

Considering all samples collected in the study, seropositive individuals were detected in 10/16 villages in Côte d’Ivoire (Djidoubay, Goulegui, Keibly, Portgenti, Sakre, Sioblooula, Tieleoula, Tienkoula, Zaipobly, and Ziriglo) and in 3/5 villages in DR Congo (Ipope, Lompole, and Nganda). The prevalence per village ranged from 0 to 8.8% (see **Table 1**); there was no statistical difference in prevalence between the villages (*p* > 0.05). Seropositivity was detected in 19 females and 15 males, in individuals that were between 3 and 81 years old, and in people with the following occupation: agriculture, sales/business persons, pupils, tailors, fishermen, and house workers. There was no statistically significant effect of gender, age, or occupation on serology status (*p*-value for all factors >0.05). The statistical model explained 8.4% of the variation in the serological status. There was no statistically significant difference between the prevalence in Côte d’Ivoire and DR Congo in any village and for all villages together (*p* > 0.05).

In comparison to previous studies, the prevalence was significantly higher (*p* = 0.002) in Côte d’Ivoire than reported for Guinea [1.2% (Klempa et al., 2010); 8/649; 95% CI 0.5–2.4%] and South Africa [1.0% (Witkowski et al., 2014); 14/1442; 95% CI 0.5–1.5%]. RT-PCR testing of 13 individuals with high IgM values did not reveal any detectable viremia within the study group.

### Health Problems within Study Population

Self-reported symptoms among the probands included problems common in African villages: 36% of the participants reported at least one type of recent health problem. Most common were malaria (*n* = 127), cough (*n* = 55), back pain (*n* = 47), and abdominal pain (*n* = 37). Four people reported fever. Of the hanta seropositive people four reported malaria, six reported cough, five reported back pain, two reported abdominal pain, and nine reported other symptoms. No hanta positive person reported fever only. Self-reported “malaria” is likely meaning symptoms that include fever. The self-reported medical information was not included into the statistical model due to the large number of different symptoms.

## Discussion

Our work shows that people in the two studied African regions carry antibodies against hantavirus, strongly suggesting their previous exposure to this pathogen. For two countries so far not investigated in this matter, seroprevalence rates of 3.9% in Côte d’Ivoire and 2.4% in DR Congo were ascertained. In recent studies performed in Guinea and in South Africa with the same antigens and methodology, seroprevalence rates of 1.2 and 1.0%, respectively, were found in the average populations (Klempa et al., 2010; Witkowski et al., 2014), while elevated hantavirus seroprevalence (4.4%) could be found among Guinean patients with uncharacterized fever (Klempa et al., 2010, 2013). Our study demonstrates a significantly higher seroprevalence of hantavirus antibodies within the major tropical rainforest region of Côte d’Ivoire (but not DR Congo) in comparison to our findings from Guinea or South Africa. This indicates that some populations living in tropical forest regions with their rich biodiversity and high potential for close contact to, e.g., wildlife hosts encounter relatively higher exposure to hantaviruses. While there is molecular evidence for the occurrence of bat- and shrew-associated hantaviruses in Côte d’Ivoire (Kang et al., 2011; Sumibcay et al., 2012), the association of these particular viruses with the human infections remains, however, to be shown.

About 97% of people above 16 years participating in the study reported consumption of rodents and other small mammals. Professional exposure seemed not to play a statistically significant role within our study group, however, occupations in agriculture and meat preparation, usually having a higher risk for hantavirus infections (Mertens et al., 2011), made up more than 70% of the probands, not including pupils. Many participants usually helped out in agriculture during harvest season, even if they work somewhere else, or they change occupation frequently. All these influencing variables might contribute to the postulated high exposure.

Among the study group, many people reported a large variety of recent health problems that are common in African villages, mainly malaria (probably meaning fever and headache), cough, back pain, and abdominal pain. On the other hand no serious health problems typical for hantavirus disease with severe course, like hemorrhages or organ failure (Krüger et al., 2011), were mentioned. Hantavirus-seropositive people did not report more health problems than the rest of the study group. In future studies a specific survey of patients with fever and hemorrhages should be a focal point for an investigation of hantavirus disease within the tropical forest biome.

Assuming a seroprevalence of 3.9 % in the entire population of Côte d’Ivoire and considering the data from Germany [seroprevalence 1.7% (Zöller et al., 1995), incidence 3.40 (Krüger et al., 2013)] and Finland [seroprevalence 5%, incidence 43.20 (Vaheri et al., 2013)], one could expect the occurrence of up to 7,000 clinical cases/year for an estimated population of ~20 millions.

The immunoassays used in the study exploit the immunodominance of hantavirus nucleocapsid protein (Meisel et al., 2006) within the infected organism. The use of antigens from African Sangassou virus, but also from Puumala and Dobrava-Belgrade viruses, within our screening increased (due to cross reactivity) the chance of detecting also hantavirus antibodies directed against still uncharacterized, indigenous viruses (Araki et al., 2001; Kucinskaite-Kodze et al., 2010). The cascade of confirmatory tests was used to exclude false positive results even though it might have decreased the sensitivity. The fact that the primary screening ELISA detected 229/982 samples as positive against Sangassou virus but only 28/982 samples positive against Puumala virus supports the hypothesis of a Sangassou-like virus being the agent responsible for the infections. Finally, the relatively low number of samples positive in all confirmatory assays makes calculations statistically difficult. In future studies the usage of antigens from additional indigenous rodent-, insectivore-, and bat-borne viruses should be forced which is hampered by restricted sequence information for novel African viruses and the complete lack of cell culture isolates of viruses except for Sangassou virus.

In all 13 IgG-positive samples found to be also IgM-positive, no viral RNA was found by RT-PCR techniques. This is not surprising as hantavirus nucleic acid can be usually detected only up to 30 days after infection under good sampling and laboratory conditions (Ettinger et al., 2012; Korva et al., 2013), while IgM can last for up to 2 years (Meisel et al., 2006; Hofmann et al., 2014).

The high proportion of samples positive in Sangassou virus-specific ELISA allows to assume that a close relative could be present in the investigated regions. The fact that six indigenous hantaviruses have been molecularly detected in recent years in Western Africa, together with our results presented here, consolidate this region as a putative hantavirus hotspot. The existence of very diverse hantaviruses hosted by different animal species within the area could lead to the emergence of new variants with high public health impact. We are convinced that more alertness should be paid to possible human hantavirus infections, particularly in the western part of Africa.

## Conflict of Interest Statement

The authors declare that the research was conducted in the absence of any commercial or financial relationships that could be construed as a potential conflict of interest.

## References

[B1] Araki K. Yoshimatsu K. Ogino M. Ebihara H. Lundkvist A. Kariwa H. (2001). Truncated hantavirus nucleocapsid proteins for serotyping Hantaan, Seoul, and Dobrava hantavirus infections. *J. Clin. Microbiol.* 39 2397–2404. 10.1128/JCM.39.7.2397-2404.2001 11427545 PMC88161

[B2] Baize S. Pannetier D. Oestereich L. Rieger T. Koivogui L. Magassouba N. (2014). Emergence of Zaire Ebola virus disease in Guinea – Preliminary report. *N. Engl. J. Med*. 371 1418–1425. 10.1056/NEJMoa1404505 24738640

[B3] de Groot R. J. Baker S. C. Baric R. S. Brown C. S. Drosten C. Enjuanes L. (2013). Middle East respiratory syndrome coronavirus (MERS-CoV): announcement of the Coronavirus Study Group. *J. Virol.* 87 7790–7792. 10.1128/JVI.01244-13 23678167 PMC3700179

[B4] Ettinger J. Hofmann J. Enders M. Tewald F. Oehme R. M. Rosenfeld U. M. (2012). Multiple synchronous outbreaks of Puumala virus, Germany, 2010. *Emerg. Infect. Dis.* 18 2010–2013. 10.3201/eid1809.111447 PMC3437711 22932394

[B5] Heinz F. X. Stiasny K. (2012). Flaviviruses and flavivirus vaccines. *Vaccine* 30 4301–4306. 10.1016/j.vaccine.2011.09.114 22682286

[B6] Hofmann J. Meier M. Enders M. Führer A. Ettinger J. Klempa B. (2014). Hantavirus disease in Germany due to infection with Dobrava-Belgrade virus genotype Kurkino. *Clin. Microbiol. Infect.* 20 O648–O655. 10.1111/1469-0691.12543 24438436

[B7] Jones P. (1994). Biodiversity in the Gulf of Guinea: an overview. *Biodivers. Conserv.* 3 772–784. 10.1007/BF00129657

[B8] Kang H. J. Kadjo B. Dubey S. Jacquet F. Yanagihara R. (2011). Molecular evolution of Azagny virus, a newfound hantavirus harbored by the West African pygmy shrew *(Crocidura obscurior*) in Côte d’Ivoire. *Virol. J.* 8:373 10.1186/1743-422X-8-373 PMC3163557 21798050

[B9] Keesing F. Belden L. K. Daszak P. Dobson A. Harvell C. D. Holt R. D. (2010). Impacts of biodiversity on the emergence and transmission of infectious diseases. *Nature* 468 647–652. 10.1038/nature09575 21124449 PMC7094913

[B10] Klempa B. Fichet-Calvet E. Lecompte E. Auste B. Aniskin V. Meisel H. (2006). Hantavirus in African wood mouse, Guinea. *Emerg. Infect. Dis.* 12 838–840. 10.3201/eid1205.051487 16704849 PMC3374458

[B11] Klempa B. Koivogui L. Sylla O. Koulemou K. Auste B. Krüger D. H. (2010). Serological evidence of human hantavirus infections in Guinea, west Africa. *J. Infect. Dis.* 201 1031–1034. 10.1086/651169 20187741

[B12] Klempa B. Koulemou K. Auste B. Emmerich P. Thomé-Bolduan C. Günther S. (2013). Seroepidemiological study reveals regional co-occurrence of Lassa- and Hantavirus antibodies in Upper Guinea, West Africa. *Trop. Med. Int. Health* 18 366–371. 10.1111/tmi.12045 23279760

[B13] Klempa B. Witkowski P. T. Popugaeva E. Auste B. Koivogui L. Fichet-Calvet E. (2012). Sangassou virus, the first hantavirus isolate from Africa, displays genetic and functional properties distinct from those of other murinae-associated hantaviruses. *J. Virol.* 86 3819–3827. 10.1128/JVI.05879-11 22278233 PMC3302504

[B14] Kortepeter M. G. Bausch D. G. Bray M. (2011). Basic clinical and laboratory features of filoviral hemorrhagic fever. *J. Infect. Dis.* 204(Suppl.) S810–S816. 10.1093/infdis/jir299 21987756

[B15] Korva M. Saksida A. Kejžar N. Schmaljohn C. Avšič-Županc T. (2013). Viral load and immune response dynamics in patients with haemorrhagic fever with renal syndrome. *Clin. Microbiol. Infect.* 19 E358–E366. 10.1111/1469-0691.12218 23573903

[B16] Krüger D. H. Figueiredo L. T. M. Song J. Klempa B. (2015). Hantaviruses – Globally emerging pathogens. *J. Clin. Virol*. 10.1016/j.jcv.2014.08.033 [Epub ahead of print]. 25453325

[B17] Krüger D. H. Schönrich G. Klempa B. (2011). Human pathogenic hantaviruses and prevention of infection. *Hum. Vaccin.* 7 685–693. 10.4161/hv.7.6.15197 21508676 PMC3219076

[B18] Krüger D. H. Ulrich R. G. Hofmann J. (2013). Hantaviruses as zoonotic pathogens in Germany. *Dtsch. Arztebl. Int.* 110 461–467. 10.3238/arztebl.2013.0461 23964302 PMC3722641

[B19] Kucinskaite-Kodze I. Petraityte-Burneikiene R. Zvirbliene A. Hjelle B. Medina R. A. Gedvilaite A. (2010). Characterization of monoclonal antibodies against hantavirus nucleocapsid protein and their use for immunohistochemistry on rodent and human samples. *Arch. Virol.* 156 443–456. 10.1007/s00705-010-0879-6 21161552 PMC8628251

[B20] McLay L. Liang Y. Ly H. (2014). Comparative analysis of disease pathogenesis and molecular mechanisms of New World and Old World arenavirus infections. *J. Gen. Virol.* 95 1–15. 10.1099/vir.0.057000-0 24068704 PMC4093776

[B21] Meisel H. Wolbert A. Razanskiene A. Marg A. Kazaks A. Sasnauskas K. (2006). Development of novel immunoglobulin G (IgG), IgA, and IgM enzyme immunoassays based on recombinant Puumala and Dobrava hantavirus nucleocapsid proteins. *Clin. Vaccine Immunol.* 13 1349–1357. 10.1128/CVI.00208-06 17021245 PMC1694442

[B22] Mertens M. Hofmann J. Petraityte-Burneikiene R. Ziller M. Sasnauskas K. Friedrich R. (2011). Seroprevalence study in forestry workers of a non-endemic region in eastern Germany reveals infections by Tula and Dobrava-Belgrade hantaviruses. *Med. Microbiol. Immunol*. 200 263–268. 10.1007/s00430-011-0203-4 21611907

[B23] Sumibcay L. Kadjo B. Gu S. H. Kang H. J. Lim B. K. Cook J. A (2012). Divergent lineage of a novel hantavirus in the banana pipistrelle (*Neoromicia nanus*) in Côte d’Ivoire. *Virol. J.* 9:34 10.1186/1743-422X-9-34 PMC3331809 22281072

[B24] Vaheri A. Henttonen H. Voutilainen L. Mustonen J. Sironen T. Vapalahti O. (2013). Hantavirus infections in Europe and their impact on public health. *Rev. Med. Virol*. 23 35–49. 10.1002/rmv.1722 22761056

[B25] Weiss S. Witkowski P. T. Auste B. Nowak K. Weber N. Fahr J. (2012). Hantavirus in Bat, Sierra Leone. *Emerg. Infect. Dis.* 18 159–161. 10.3201/eid1801.111026 22261176 PMC3310113

[B26] Witkowski P. T. Klempa B. Ithete N. L. Auste B. Mfune J. K. E. Hoveka J. (2014). Hantaviruses in Africa. *Virus Res.* 187 34–42. 10.1016/j.virusres.2013.12.039 24406800

[B27] Wolfe N. D. Dunavan C. P. Diamond J. (2007). Origins of major human infectious diseases. *Nature* 447 279–283. 10.1038/nature05775 17507975 PMC7095142

[B28] Zöller L. Faulde M. Meisel H. Ruh B. Kimmig P. Schelling U. (1995). Seroprevalence of hantavirus antibodies in Germany as determined by a new recombinant enzyme immunoassay. *Eur. J. Clin. Microbiol. Infect. Dis.* 14 305–313. 10.1007/BF02116523 7649193

