# Supplementary material for: Human seroprevalence indicating hantavirus infections in tropical rainforests of Côte d’Ivoire and Democratic Republic of Congo
Source: Front Microbiol. 2015 May 21;6:518. doi: 10.3389/fmicb.2015.00518 (PMC4439549; doi:10.3389/fmicb.2015.00518)
Supplement: Supplementary file 1 [file Table_1.DOCX]

**TABLE S1.** **Outcome of serodiagnostic assays for the 34 persons tested positive for hantavirus antibodies.** Abbreviations: n.d. = not done (because of low sample volume; yet seropositivity criteria were fulfilled)

| **Sample** | | | **Screening assays** | | | **Confirmation assays** | | | | | | | | | |
| --- | --- | --- | --- | --- | --- | --- | --- | --- | --- | --- | --- | --- | --- | --- | --- |
| **ID** | **Village** | **Country** | **ELISA** | | | **Blot** | | | | | | **IFA (titer)** | | | |
|  |  |  | **IgG** | | | **in house** | **Mikrogen** | | | | | **in house** | | | |
|  |  |  | **SANGV** | **PUUV** | **DOBV** | **SANGV** | **Htnv+Puuv** | **PUUV** | **HTNV** | **DOBV** | **SEOV** | **SANGV** | **PUUV** | **DOBV** | **HTNV** |
| **DJI038** | **Djidoubaye** | **IC** | **+** | **-** | **+** | **+** | **-** | **-** | **-** | **+** | **-** | **≥20** | **-** | **-** | **≥20** |
| **GUL013** | **Goulegui Beoue** | **IC** | **+** | **-** | **-** | **+** | **nd** | **nd** | **nd** | **nd** | **nd** | **≥20** | **-** | **-** | **-** |
| **I416** | **Ipope** | **DRC** | **+** | **-** | **+** | **nd** | **+** | **-** | **+** | **+** | **-** | **-** | **≥20** | **-** | **-** |
| **IEN005** | **Tienkoula** | **IC** | **-** | **+** | **-** | **-** | **-** | **+** | **-** | **-** | **-** | **-** | **≥20** | **-** | **-** |
| **IEN023** | **Tienkoula** | **IC** | **-** | **-** | **+** | **+** | **nd** | **nd** | **nd** | **nd** | **nd** | **-** | **-** | **-** | **≥20** |
| **KEI004** | **Keibly** | **IC** | **+** | **-** | **-** | **+** | **nd** | **nd** | **nd** | **nd** | **nd** | **≥20** | **-** | **-** | **-** |
| **KEI006** | **Keibly** | **IC** | **+** | **-** | **+** | **+** | **-** | **-** | **-** | **+** | **-** | **-** | **≥20** | **-** | **-** |
| **KEI018** | **Keibly** | **IC** | **+** | **-** | **-** | **+** | **nd** | **nd** | **nd** | **nd** | **nd** | **≥20** | **-** | **-** | **-** |
| **KEI023** | **Keibly** | **IC** | **+** | **-** | **-** | **+** | **nd** | **nd** | **nd** | **nd** | **nd** | **≥20** | **≥20** | **-** | **≥20** |
| **KEI027** | **Keibly** | **IC** | **+** | **-** | **-** | **+** | **nd** | **nd** | **nd** | **nd** | **nd** | **-** | **≥20** | **-** | **-** |
| **KEI031** | **Keibly** | **IC** | **+** | **-** | **-** | **+** | **nd** | **nd** | **nd** | **nd** | **nd** | **-** | **-** | **≥20** | **-** |
| **KEI066** | **Keibly** | **IC** | **-** | **-** | **+** | **-** | **-** | **-** | **-** | **+** | **-** | **-** | **≥20** | **-** | **-** |
| **L031** | **Lompole** | **DRC** | **+** | **-** | **+** | **-** | **+** | **-** | **+** | **+** | **-** | **≥20** | **-** | **-** | **≥20** |
| **L063** | **Lompole** | **DRC** | **+** | **-** | **+** | **-** | **+** | **-** | **+** | **+** | **+** | **80** | **-** | **80** | **80** |
| **L110** | **Lompole** | **DRC** | **+** | **+** | **+** | **-** | **+** | **+** | **-** | **-** | **-** | **-** | **≥20** | **-** | **-** |
| **L467** | **Lompole** | **DRC** | **-** | **+** | **-** | **nd** | **+** | **+** | **-** | **-** | **-** | **-** | **80** | **-** | **-** |
| **N341** | **Nganda** | **DRC** | **+** | **-** | **+** | **nd** | **-** | **+** | **+** | **+** | **-** | **40** | **-** | **40** | **-** |
| **N379** | **Nganda** | **DRC** | **+** | **-** | **+** | **nd** | **-** | **-** | **+** | **+** | **-** | **40** | **≥160** | **160** | **80** |
| **POR008** | **Portgentil** | **IC** | **+** | **-** | **-** | **+** | **-** | **-** | **-** | **-** | **-** | **-** | **≥20** | **-** | **-** |
| **SAK002** | **Sakre** | **IC** | **+** | **-** | **+** | **+** | **nd** | **nd** | **nd** | **nd** | **nd** | **20** | **-** | **≥20** | **≥20** |
| **SAK017** | **Sakre** | **IC** | **+** | **-** | **-** | **+** | **nd** | **nd** | **nd** | **nd** | **nd** | **-** | **≥20** | **-** | **-** |
| **SAK025** | **Sakre** | **IC** | **+** | **-** | **-** | **+** | **nd** | **nd** | **nd** | **nd** | **nd** | **-** | **-** | **≥20** | **≥20** |
| **SAK042** | **Sakre** | **IC** | **+** | **-** | **+** | **+** | **+** | **-** | **+** | **+** | **+** | **-** | **-** | **-** | **≥20** |
| **SAK059** | **Sakre** | **IC** | **+** | **-** | **-** | **+** | **nd** | **nd** | **nd** | **nd** | **nd** | **-** | **-** | **-** | **≥20** |
| **SAK072** | **Sakre** | **IC** | **+** | **-** | **-** | **+** | **nd** | **nd** | **nd** | **nd** | **nd** | **-** | **≥20** | **≥20** | **≥20** |
| **SIO013** | **Sioblooula** | **IC** | **+** | **+** | **nd** | **+** | **nd** | **nd** | **nd** | **nd** | **nd** | **-** | **-** | **-** | **≥20** |
| **SIO033** | **Sioblooula** | **IC** | **+** | **-** | **+** | **+** | **nd** | **nd** | **nd** | **nd** | **nd** | **-** | **≥20** | **-** | **≥20** |
| **SIO034** | **Sioblooula** | **IC** | **+** | **-** | **+** | **+** | **nd** | **nd** | **nd** | **nd** | **nd** | **-** | **-** | **-** | **≥20** |
| **TIE030** | **Tieleoula** | **IC** | **+** | **nd** | **nd** | **+** | **nd** | **nd** | **nd** | **nd** | **nd** | **-** | **-** | **-** | **≥20** |
| **TIE036** | **Tieleoula** | **IC** | **+** | **+** | **+** | **+** | **nd** | **nd** | **nd** | **nd** | **nd** | **-** | **≥20** | **-** | **-** |
| **ZAI020** | **Zaipobly** | **IC** | **+** | **nd** | **+** | **+** | **+** | **-** | **+** | **+** | **-** | **≥20** | **-** | **-** | **-** |
| **ZAI026** | **Zaipobly** | **IC** | **+** | **nd** | **nd** | **+** | **nd** | **nd** | **nd** | **nd** | **nd** | **≥20** | **-** | **-** | **-** |
| **ZAI031** | **Zaipobly** | **IC** | **+** | **nd** | **nd** | **+** | **nd** | **nd** | **nd** | **nd** | **nd** | **-** | **-** | **≥20** | **-** |
| **ZIR014** | **Ziriglo** | **IC** | **+** | **-** | **-** | **+** | **nd** | **nd** | **nd** | **nd** | **nd** | **-** | **-** | **≥20** | **-** |
